# Supplementary material for: Acute opioid responses are modulated by dynamic interactions of Oprm1 and Fgf12
Source: eLife. 2026 Jul 29;14:RP108845. doi: 10.7554/eLife.108845 (PMC13421559; doi:10.7554/eLife.108845)
Supplement: Peer review history

# Editor report: eLife Assessment

**DOI:** 10.7554/eLife.108845.3.sa0

**Authors:** Jonathan Flint

**Affiliations:** University of California, Los Angeles United States

**Keywords:** Compelling, Fundamental

This study integrates large-scale behavioral, genetic, and molecular analyses in animal models to investigate morphine response. Utilizing high-quality, time-series Quantitative Trait Loci (QTL) mapping, the work provides **compelling** evidential support for novel, time-dependent genetic interactions (epistasis). A **fundamental** result of this rigorous analysis is the discovery of a novel Oprm1-Fgf12-MAPK signaling pathway, which offers new insights into the mechanisms of opioid sensitivity.

# Referee report: Reviewer #1 (Public review)

**DOI:** 10.7554/eLife.108845.3.sa1

[Editors' note: this version has been assessed by the Reviewing Editor without further input from the original reviewers. The authors have appropriately addressed the comments raised in the previous round of review.]

Summary:

The study by Lemen et al. represents a comprehensive and unique analysis of gene networks in rat models of opioid use disorder, using multiple strains and both sexes. It provides a time-series analysis of Quantitative Trait Loci (QTLs) in response to morphine exposure.

Strengths:

A key finding is the identification of a previously unknown morphine-sensitive pathway involving Oprm1 and Fgf12, which activates a cascade through MAPK kinases in D1 medium spiny neurons (MSNs). Strengths include the large-scale, multi-strain, sex-inclusive design, the time-series QTL mapping provides dynamic insights, and the discovery of an Oprm1-Fgf12-MAPK signaling pathway in D1 MSNs, which is novel and relevant.

# Referee report: Reviewer #2 (Public review)

**DOI:** 10.7554/eLife.108845.3.sa2

Summary:

This highly novel and significant manuscript re-analyzes behavioral QTL data derived from morphine locomotor activity in the BXD recombinant inbred panel. The combination of interacting behavioral-pharmacology (morphine and naltrexone) time course data, high-resolution mouse genetic analyses, genetic analysis of gene expression (eQTLs), cross-species analysis with human gene expression and genetic data, and molecular modeling approaches with Bayesian network analysis produces new information on loci modulating morphine locomotor activity.

Furthermore, the identification of time-wise epistatic interactions between the Oprm1 and Fgf12 loci is highly novel and points to methodological approaches for identifying other epistatic interactions using animal model genetic studies.

Strengths:

(1) Use of state-of-the art genetic tools for mapping behavioral phenotypes in mouse models.

(2) Adequately powered analysis incorporating both sexes and time course analyses.

(3) Detection of time and sex-dependent interactions of two QTL loci modulating morphine locomotor activity.

(4) Identification of putative candidate genes by combined expression and behavioral genetic analyses.

(5) Use of Bayesian analysis to model causal interactions between multiple genes and behavioral time points.

Appraisal:

The authors largely succeeded in reaching goals with novel findings and methodology.

Significance of Findings:

This study will likely spur future direct experimental studies to test hypotheses generated by this complex analysis. Additionally, the broad methodological approach incorporating time course genetic analyses may encourage other studies to identify epistatic interactions in mouse genetic studies.

# Referee report: Reviewer #3 (Public review)

**DOI:** 10.7554/eLife.108845.3.sa3

Summary:

This is a clearly written paper that describes the reanalysis of data from a BXD study of the locomotor response to morphine and naloxone. The authors detect significant loci and an epistatic interaction between two of those loci. Single-cell data from outbred rats is used to investigate the interaction. The authors also use network methods and incorporate human data into their analysis.

Strengths:

One major strength of this work is the use of granular time-series data, enabling the identification of time-point-specific QTL. This allowed for the identification of an additional, distinct QTL (the Fgf12 locus) in this work compared to previously published analysis of these data, as well as the identification of an epistatic effect between Oprm1 (driving early stages of locomotor activation) and Fgf12 (driving later stages).

# Author comment: Author response

**DOI:** 10.7554/eLife.108845.3.sa4

**Authors:** Paige Lemen, Yanning Zuo, Alexander S Hatoum, Price E Dickson, Guy Mittleman, Arpana Agrawal, Benjamin C Reiner, Wade Berrettini, David George Ashbrook, Mustafa Hakan Gunturkun, Xusheng Wang, Megan K Mulligan, Caleb Brown, Eric J Nestler, Francesca Telese, Robert Williams, Hao Chen

**Affiliations:** University of Tennessee Health Science Center, Memphis, TN 38163 USA Memphis United States; Department of Psychiatry, University of California, San Diego San Diego United States; Washington University, St. Louis, MO 63130, USA St. Louis United States; Marshall University, Huntington, WV 25755, USA Huntington United States; Ball State University, Muncie IN 47306, USA Muncie United States; Washington University School of Medicine Washington United States; University of Pennsylvania, Philadelphia, PA 19104, USA Philadelphia United States; University of Pennsylvania, Philadelphia, PA 19104, USA Philadelphia United States; University of Edinburgh Edinburgh United Kingdom; University of Tennessee Health Science Center, Memphis, TN 38163 USA Memphis United States; University of Tennessee Health Science Center, Memphis, TN, USA Memphis United States; University of Tennessee Health Science Center Memphis United States; Icahn School of Medicine at Mount Sinai New York United States; Mt. Sinai New York United States; Department of Psychiatry, University of California, San Diego San Diego United States; University of Tennessee Health Science Center Memphis United States; University of Tennessee Health Science Center Memphis United States

The following is the authors’ response to the original reviews.

**Public Reviews:**

**Reviewer #1 (Public review):**

Summary:

The study by Lemen et al. represents a comprehensive and unique analysis of gene networks in rat models of opioid use disorder, using multiple strains and both sexes. It provides a time-series analysis of Quantitative Trait Loci (QTLs) in response to morphine exposure.

Strengths:

A key finding is the identification of a previously unknown morphine-sensitive pathway involving Oprm1 and Fgf12, which activates a cascade through MAPK kinases in D1 medium spiny neurons (MSNs). Strengths include the large-scale, multi-strain, sex-inclusive design, the time-series QTL mapping provides dynamic insights, and the discovery of an Oprm1-Fgf12-MAPK signaling pathway in D1 MSNs, which is novel and relevant.

Weaknesses:

(1) The proposed involvement of Nav1.2 (SCN2A) as a downstream target of the Oprm1-Fgf12 pathway requires further analysis/evidence. Is Nav1.2 (SCN2A) expressed in D1 neurons?

The authors mentioned that SCN8A (Nav1.6) was tested as a candidate mediator of Oprm1-Fgf12 loci and variation in locomotor activity. However, the proposed model supports SCN2A as a target rather than SCN8A. This is somewhat unexpected since SCN8A is highly abundant in MSN.

Can the authors provide expression data for SCN2A, Oprm1, and Fgf12 in D1 vs. D2 MSNs?

**Author response image 1.** 

We generated Author response image 1 to show both Scn2a and Scn8a are ubiquitously expressed in MSN and GABAergic neurons.

(2) The authors should consider adding a reference to FGF12 in Schizophrenia (PMC8027596) in the Introduction.

This is a relevant reference. We have cited it in the discussion section instead of introduction because we felt that is more relevant.

(3) There is recent evidence supporting the druggability of other intracellular FGFs, such as FGF14 (PMC11696184) and FGF13 (PMC12259270), through their interactions with Nav channels. What are the implications of these findings for drug discovery in the context of the present study? Could FGF12 be considered a potential druggable therapeutic target for opioid use disorder (OUD)?

The recent success in targeting FGF14 and FGF13 protein-protein interactions with sodium channels suggests that FGF12 could indeed be a druggable target for OUD. We have added a section to the Discussion exploring the potential for developing small-molecule modulators of the FGF12-Nav interface as a novel therapeutic strategy.

**Reviewer #2 (Public review):**

Summary:

This highly novel and significant manuscript re-analyzes behavioral QTL data derived from morphine locomotor activity in the BXD recombinant inbred panel. The combination of interacting behavioral-pharmacology (morphine and naltrexone) time course data, high-resolution mouse genetic analyses, genetic analysis of gene expression (eQTLs), cross-species analysis with human gene expression and genetic data, and molecular modeling approaches with Bayesian network analysis produces new information on loci modulating morphine locomotor activity.

Furthermore, the identification of time-wise epistatic interactions between the Oprm1 and Fgf12 loci is highly novel and points to methodological approaches for identifying other epistatic interactions using animal model genetic studies.

Strengths:

(1) Use of state-of-the art genetic tools for mapping behavioral phenotypes in mouse models.

(2) Adequately powered analysis incorporating both sexes and time course analyses.

(3) Detection of time and sex-dependent interactions of two QTL loci modulating morphine locomotor activity.

(4) Identification of putative candidate genes by combined expression and behavioral genetic analyses.

(5) Use of Bayesian analysis to model causal interactions between multiple genes and behavioral time points.

Weaknesses:

(1) There is a need for careful editing of the text and figures to eliminate multiple typographical and other compositional errors.

We have performed a thorough review of the manuscript and corrected typographical errors, including "ddactivates" and other compositional issues.

(2) There are multiple examples of overstating the possible significance of results that should be corrected or at least directly pointed out as weaknesses in the Discussion. These include:

(a) Assumption that the Oprm1 gene is the causal candidate gene for the major morphine locomotor Chr10 QTL at the early time epochs. Oprm1 is 400,000 bp away from the support interval of the Mor10a QTL locus, and there is no mention as to whether the Oprm1 mRNA eQTL overlaps with Mor10a.

We have clarified this in the text. While *Oprm1* is located proximal to the peak, its massive size and the presence of a strong mRNA cis-eQTL in the NAc and hippocampus that precisely overlaps with the *Mor10a* QTL support interval provide robust evidence for its candidacy. We have added this detail to the Results section.

(b) Although the Bayesian analysis of possible complex interactions between Oprm1, Fgf12, other interacting genes, and behaviors is very innovative and produces testable hypotheses, a more straightforward mediation analysis of causal relationships between genotype, gene expression, and phenotype would have added strength to the arguments for the causal role of these individual genes.

We agree that mediation analysis would be a valuable addition. We revised the Results section to acknowledge that while the Bayesian network provides a comprehensive causal hypothesis, future studies employing formal mediation analysis could further strengthen these individual gene-to-behavior links.

(c) The GWAS data analysis for Oprm1 and Fgf12 is incomplete in not mentioning actual significance levels for Oprm1 and perhaps overstating the nominal significance findings for Fgf12.

We have updated the manuscript to include the specific significance levels for the human GWAS findings related to *Oprm1* and *Fgf12*. We have clarified that the *OPRM1* variant rs1799971 reached genome-wide significance (OR = 1.046, *p* = 4.92 × 10^-9^). Furthermore, we have ensured that the findings for *FGF12* are described as nominally significant to avoid any overstatement of the results. For example, we now specify that the top *FGF12* SNP rs1553460 achieved nominal significance (OR = 1.015, *p* = 0.021). The Results and Discussion sections have been revised to reflect these precise statistical values.

Appraisal:

The authors largely succeeded in reaching goals with novel findings and methodology.

Significance of Findings:

This study will likely spur future direct experimental studies to test hypotheses generated by this complex analysis. Additionally, the broad methodological approach incorporating time course genetic analyses may encourage other studies to identify epistatic interactions in mouse genetic studies.

**Reviewer #3 (Public review):**

Summary:

This is a clearly written paper that describes the reanalysis of data from a BXD study of the locomotor response to morphine and naloxone. The authors detect significant loci and an epistatic interaction between two of those loci. Single-cell data from outbred rats is used to investigate the interaction. The authors also use network methods and incorporate human data into their analysis.

Strengths:

One major strength of this work is the use of granular time-series data, enabling the identification of time-point-specific QTL. This allowed for the identification of an additional, distinct QTL (the Fgf12 locus) in this work compared to previously published analysis of these data, as well as the identification of an epistatic effect between Oprm1 (driving early stages of locomotor activation) and Fgf12 (driving later stages).

Weaknesses:

(1) What criteria were used to determine whether the epistatic interaction was significant? How many possible interactions were explored?

By design we only tested for epistasis between the Oprm1 and the Fgf12 loci—a single test of a non-linear interaction. As such there is no correction for multiple tests and no need for permutation. In other words the “nominal” P value in this case is the only relevant P value. We have added this clarification in the Results and Methods.

(2) Results are presented for males and females separately, but the decision to examine the two sexes separately was never explained or justified. Since it is not standard to perform GWAS broken down by sex, some initial explanation of this decision is needed. Perhaps the discussion could also discuss what (if anything) was learned as a result of the sex-specific analysis. In the end, was it useful?

We chose to analyze sexes separately AND jointly due to significant sex differences and sex by strain interactions in locomotion data. This rationale has been added to the results section. We also discussed sex-specific results in the revision.

(3) The confidence intervals for the results were not well described, although I do see them in one of the tables. The authors used a 1.5 support interval, but didn't offer any justification for this decision. Is that a 95% confidence interval? If not, should more consideration have been given to genes outside that interval? For some of the QTLs that are not the focus of this paper, the confidence intervals were very large (>10 Mb). Is that typical for BXDs?

The 1.5 LOD support interval is a standard metric for most QTL mapping studies, and does correspond approximately to a 95% confidence or support interval. Large intervals are common in BXD studies when effect sizes are moderate or recombination density is lower in specific regions. We have clarified the use of the 1.5 LOD interval in the Results section.

**Recommendations for the authors:**

**Reviewer #1 (Recommendations for the authors):**

In the vast majority of the figures, the text is too small to read.

We have adjusted the font size in most of the figures.

**Reviewer #2 (Recommendations for the authors):**

(1) There is a need for careful editing of the text and figures to eliminate multiple typographical and other compositional errors. Examples of these include:

(a) Figure 2E&F lacks identification of Oprm1 as the gene for cis-eQTL studies.

(b) Figure 2H is fairly uninterpretable given the small font sizes. It should be excluded, put as a supplemental figure, or reconfigured to highlight the most important findings in a more legible manner.

(c) Figure 4b: columns in the table need to be identified by a header row.

We thank the reviewer for these comments and have addressed them in the revised version.

Oprm1 is now labeled in Figure 2E and 2F, Figure 2G and 2H is now moved to the Supplementary material. And a header row is added to the table in Figure 4b.

**Reviewer #3 (Recommendations for the authors):**

Abstract

(1) For the abstract, it might be simpler to name the alleles as "the C57BL/6J allele", etc., since B allele will confuse people unfamiliar with mouse nomenclature.

It is critical to not confound the organism known as C57BL/6J with the genotype, allele, or haplotype that a mouse happens to inherit. Diverse types of mice inherit reference alleles but they may be only very distantly related the C57BL/6J strain. And even the C57BL/6J strain is a moving target that accumulates mutations that are not even consider reference. For example the mutation in Gabra2 of C57BL/6J is a de novo mutation that is not carried by many of the BXD strains since this mutation happened in JAX foundation stock after the BXDs were first established by Dr. Ben Taylor in the 1970s.

The convention is to refer to mouse strains by one string and RRID, the abbreviation of that strain by a common code (often B6), and the abbreviation of the allele, genotype, or haplotype by the italic letter *B*. This has been the recommendation of the Mouse Nomenclature Committee (on which one of the authors has been a member) for well over 50 years.

(2) I wondered if "also associated with a high B allele" could be reworded somehow; I had to re-read that sentence several times.

This sentence has been reworded for clarity.

(3) Parts of the abstract are written in the present tense, but then it switches to past ("we generated" but then "a Bayesian network analysis supports...").

We have thoroughly revised the abstract. Following standard scientific writing conventions, we now utilize the past tense to describe the specific experimental actions and results of this study. We have maintained the present tense for established biological facts and the broader significance of the findings.

(4) While the -log(p) values are all impressive, the abstract should indicate what threshold is used for genome-wide significance and how that threshold was obtained.

We have added the significance threshold to the Abstract.

(5) Do the details of the MAP kinase cascade need to be explained in the abstract? It feels like a lot of detail for an abstract and represents one of the most speculative aspects of the paper. Maybe just say you identified a possible network, but save the details for the main paper.

This is a valid suggestion. We removed the specific MAP kinase from the abstract.

Introduction

(1) You could add a sentence explaining why using an LMM (GEMMA) was an improvement over the prior analysis.

We have added a sentence explaining that GEMMA improves mapping power and better controls for population structure compared to previous methods.

(2) When mentioning Philips 2010, you could indicate that it identified Oprm1. This might be easier than "In addition to Oprm1" which confused me at first because it had not been mentioned before, so 'in addition' was jarring.

We have revised the text to state that Philip et al. (2010) originally identified the *Oprm1* locus.

Results

(1) There are additional instances of the tense switching between past and present in the results section.

We have standardized the tenses in the Results section.

(2) "Ostn, Uts2d, Ccdc50, Gm10823, Fgf12, and Mb21d2" - before giving arguments for fgf12, can you clarify if there are coding variants or eQTLs for any of these genes?

We have added a statement clarifying the coding variants for other genes in this interval and highlighting their eQTL status.

(3) "a total number of 4,495 high-quality nuclei transcriptomes". Consider removing the word "number".

Removed.

(4) "approximately 6 males and 6 females" - could you point the reader to a supplementary table that has the exact number of individuals at the end of this sentence?

The exact number of mice used in each of the BXD strains is not recorded in the original publication by Philip et al., with only mean and max was given. We have clarified that 6 is the average.

(5) "computed using a subset" - please explain how you selected this subset I assumed LD pruning, but why not be explicit. How many SNPs/markers were there originally, and how many are retained?

We have specified that the subset of markers was selected via LD pruning to represent the genetic diversity of the BXDs.

(6) A few words about how the significant threshold was obtained (permutation?) are needed.

We have clarified that the significance threshold was obtained through 1,000 permutations.

(7) Some of the GWAS results are presented for males and females separately (as well as combined). This is not typical, and so maybe a sentence explaining why the authors thought there might be sex specific GWAS results would be warranted.

The rationale for sex-specific analysis is provided in the results section (significant sex difference and sex by strain interaction)

(8) The correlation between the sexes of 0.68 could be evidence that there are sex-specific genetic effects, but could it also just be due to increased noise as you reduce sample size? What is the confidence interval for that number? Does it include 1? Or 0? If you randomly split the dataset, rather than splitting on the basis of sex, would you obtain higher correlations? The idea of sex differences is interesting, but a bit more work is needed to clarify these concerns.

The correlation of 0.68 (95% CI: 0.52–0.79) significantly excludes both 0 and 1. The drop from r = ~0.86 at earlier intervals suggests a biological shift rather than noise due to sample size, as n remains constant (n = ~ 6 /sex/strain) across all time points. This divergence is driven by sex-specific genetic modifiers, such as the *Fgf12* locus, which is more than twice as strong in females (LOD 10.6) as in males (LOD 4.3). We have addressed this in the revision.

(9) Maybe I missed it, but how did you determine the threshold for significance for the epistatic interaction? Could you also clearly indicate how many possible cases of epistasis were examined/considered, since that dictates the correction for multiple testing.

We only tested the interaction between the *Fgf12* and the *Oprm* loci.

(10) "To further examine whether Oprm1 and Fgf12 were co-expressed in the same cells of the NAc," can you first give an indication as to why you looked in NAc versus other brain areas you might have considered?

We have added a sentence explaining that the NAc was chosen due to its central role in opioid reward and the observed strain differences in dopamine release in this region.

(11) "...from every cell type conveyed a weak but significant positive correlation (r = 0.08, p = 1.8e-8) between the expression of Oprm1 and Fgf12 (Figure 7e). When we performed Pearson's correlation analysis within each individual cell cluster, only D1-MSN-3 had a significant positive correlation (r = 0.35, p = 6.1e-8, Figure 7f). In contrast, D1-MSN-2 had a significantly weak negative correlation (r = -0.12, p = 0.02, Figure 7g)." Can you explain why these correlations are relevant? What hypothesis are you testing?

We have clarified that these correlations were used to test the hypothesis that *Oprm1* and *Fgf12* are co-expressed and potentially co-regulated within the same neuronal subtype to support their epistatic interaction.

(12) "After the morphine locomotion tests were complete," can you give a specific timepoint? Like, was it exactly 180 minutes after the morphine injection?

We have specified that naloxone was injected exactly 180 minutes after the morphine injection.

(13) I appreciate the desire to relate the results of this paper to human GWAS results; however, I don't feel there is much worth discussing beyond the Oprm1 finding. Therefore, I would suggest removing this from the results section and instead just making it a discussion topic. The results presented are clearly the weakest part of this paper, and I personally think it is a shame to end the results section with something that is not very informative. But I suspect the authors may wish to retain this section, and I leave that decision to them and the editor.

We have retained this section but moved some of the more speculative human data discussion to the Discussion section as suggested.

Discussion

(1) Typo "deactivates".

Corrected to "activates".

(2) The last sentence in the first paragraph again discusses the comparison to humans; I would remove this.

That sentence is condensed.

(3) "These data indicate that Oprm1 is a strong candidate gene for the Chr 10 locus associated with morphine-induced locomotion response." I would remind them of the eQTL for Oprm1 since this is a key piece of evidence supporting this gene as a candidate.

We have added a reminder of the overlapping mRNA cis-eQTL for Oprm1.

(4) "It is likely that differences in morphine-induced dopamine release are involved in the highly variable locomotor responses to morphine across the BXD family." I agree this might be true, but since you have no evidence to support this claim, is it worth mentioning at all?

We have rephrased this as a hypothesis or cited relevant literature supporting this link in parental strains.

(5) Could you include a sentence or two about why Philip 2010 didn't find Fgf12? Lack of markers? The difference between an LM and an LMM?

We have added an explanation that the use of a high-density WGS-based marker set and the LMM (GEMMA) allowed for the detection of this novel locus that was previously missed.

(6) Section titled "Cell-type specific gene expression in NAc". While this is interesting, you might also want to remind the reader that epistatic interactions do not necessarily require the genes to be expressed in the same cell or for their gene products to physically interact.

We have added this caveat to the Discussion.

(7) I think the Bayesian network section is not very strong. For example, they did not compare the results for their two chosen genes to the results they might have obtained if they had chosen other genes from their QTL intervals. My guess is that those other genes might have also produced results that were equally convincing. I'm not asking them to do that, but it reflects the risk of false positive results when taking an approach like this. Nevertheless, I am guessing the authors would prefer to include this section.

We appreciate the reviewer pointing out this possibility and agree with this concern. We have added a statement acknowledging the risk of false positives in Bayesian modeling in this context and noting that these findings are intended as testable hypotheses

Methods

(1) How were the 2 HS rats selected? I had the impression that Dr. Telese's lab had access to snRNA-seq data from more than 2 HS rats.

We have clarified that these rats were selected based on their addiction-like behavior phenotypes from a larger cohort.

(2) I didn't look back, but did the main paper point out that the rats are treated with oxycodone rather than morphine?

We have clarified this distinction in the Methods section.
